# How Kinesin-1 Utilize the Energy of Nucleotide: The Conformational Changes and Mechanochemical Coupling in the Unidirectional Motion of Kinesin-1

**DOI:** 10.3390/ijms21186977

**Published:** 2020-09-22

**Authors:** Jingyu Qin, Hui Zhang, Yizhao Geng, Qing Ji

**Affiliations:** 1 College of Education, Shanghai Normal University, Shanghai 200234, China; qinjingyu@shnu.edu.cn; 2 School of Science, Hebei University of Technology, Tianjin 300401, China; zhanghui@hebut.edu.cn; 3 Institute of Biophysics, Hebei University of Technology, Tianjin 300401, China

**Keywords:** Kinesin-1, nucleotide, microtubule, mechanochemical coupling, neck linker, conformational change

## Abstract

Kinesin-1 is a typical motile molecular motor and the founding member of the kinesin family. The most significant feature in the unidirectional motion of kinesin-1 is its processivity. To realize the fast and processive movement on the microtubule lattice, kinesin-1 efficiently transforms the chemical energy of nucleotide binding and hydrolysis to the energy of mechanical movement. The chemical and mechanical cycle of kinesin-1 are coupled to avoid futile nucleotide hydrolysis. In this paper, the research on the mechanical pathway of energy transition and the regulating mechanism of the mechanochemical cycle of kinesin-1 is reviewed.

## 1. Introduction

**Figure 1:**
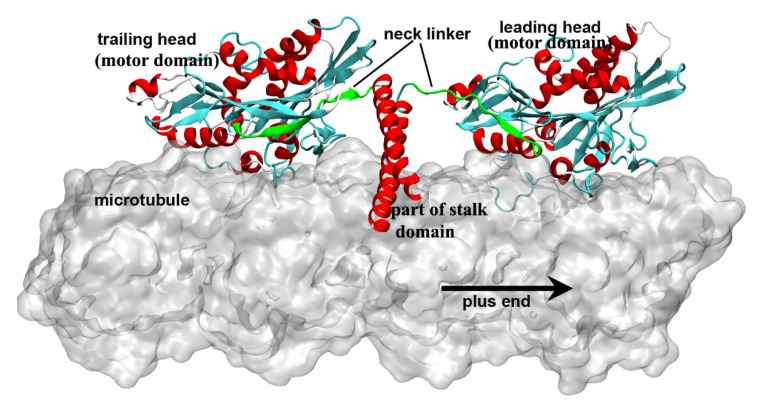
Two motor domains of kinesin-1 bind to the microtubule lattice simultaneously. The neck linkers of the kinesin-1 dimer are colored in green. The leading head is in the nucleotide-free state and has an undocked neck linker, which points to the minus end of the microtubule. The trailing head is in the ADP·Pi/ADP-bound state and has a plus-end pointed neck linker. This figure was produced using Discovery studio 3.5 visualizer.

Kinesin is a molecular walking motor protein inside cells. The size of kinesin is smaller than the other two members of motor proteins, myosin and dynein. The kinesin proteins can be divided into 14 subfamilies according to their structural and functional similarities (from kinesin-1 to kinesin-14) [1,2,3,4]. The kinesin-1 subfamily (also called conventional kinesin) is the founding member of the kinesin family [5,6] and mainly exists in the nerve axons to transport membranous organelles along microtubule lattice. Different from the kinesin-3 (monomer, but can also form a dimer [7]) and the kinesin-5 (tetramer) subfamily, the members of kinesin-1 form a dimer structure in vivo to “walk” toward the microtubule’s plus end. The entire structure of kinesin-1 can be mainly divided into three domains, i.e., the motor domain, the tail domain and the stalk domain (the motor domain and a part of stalk domain of kinesin-1 are shown in Figure 1). The motor domain (also called motor head), which contains the nucleotide-binding and microtubule-binding sites, is highly conserved among the kinesin family. The tail domain of kinesin-1 is used to bind with the “cargo”. Kinesin-1 proteins have different tail domains, which can bind the light chain to interact with different cargos [8]. The motor domain and the tail domain are connected by a single long α-helix, which is called the stalk domain. The two stalk domains of two kinesin-1 monomers coil together to form a coiled-coil structure and constitute a functional dimer. It is worthwhile to note that ~14 residues constitute the neck linker of kinesin-1, which connects the motor domain and the stalk domain. The conformational changes of the neck linker in different nucleotide-binding states are the key processes in the walking movement of kinesin-1 [9,10,11,12,13]. Because the motor domain locates in the N-terminal part of the protein, kinesin-1 belongs to the N-type kinesin. Kinesins with the motor domain located in the middle and the C-terminal part of the protein are the M-type and C-type, respectively. The walking directionality of kinesin varies with different locations of the motor domain. N-type kinesins (most members of the kinesin family) walk toward the plus end of the microtubule (some of the kinesin-5 proteins with N-terminal motor domain show bidirectional motility, as reviewed in Ref. [14]). In contrast, the C-type kinesin (mainly kinesin-14 subfamily [15]) walks toward the minus end of the microtubule. The M-type kinesin (mainly kinesin-13 subfamily) is relatively special because it takes one-dimensional diffusion toward the two ends of the microtubule [16,17,18,19].

The kinesin-1 dimer walks along a single protofilament of the microtubule in a hand-over-hand manner. There are some noteworthy features of kinesin-1 walking movement: (1) Kinesin-1 can transform chemical energy of the adenosine triphosphate (ATP) binding and hydrolysis to mechanical energy of the walking along the microtubule with a cargo. (2) The chemical cycle and the mechanical cycle of kinesin-1 are highly coupled to ensure only one ATP molecule is consumed in one step [20,21]. The futile ATP hydrolysis rarely happens in kinesin-1 normal walking process. (3) The microtubule not only provides the track for the motility of kinesin-1 but also directly participates in the regulation of the kinesin-1 chemical cycle. The microtubule can catalyze the release of adenosine diphosphate (ADP), which is the product of ATP hydrolysis. In this way, the mechanochemical process of kinesin-1 is dramatically accelerated. The key process of energy transformation is from ATP entering the nucleotide-binding pocket to the docking movement of the neck linker, which pulls the other motor domain to the next binding site on the microtubule. How the conformational changes induced by the ATP binding can transmit to the neck linker region and finally drive the neck linker docking is an essential question in the walking mechanism of kinesin-1. In this paper, research on the conformational changes from the ATP binding to the neck liner docking and the coupling of mechanochemical cycle of kinesin-1 is reviewed.

## 2. Interactions Between ATP Molecule and Motor Domain of Kinesin-1

**Figure 2:**
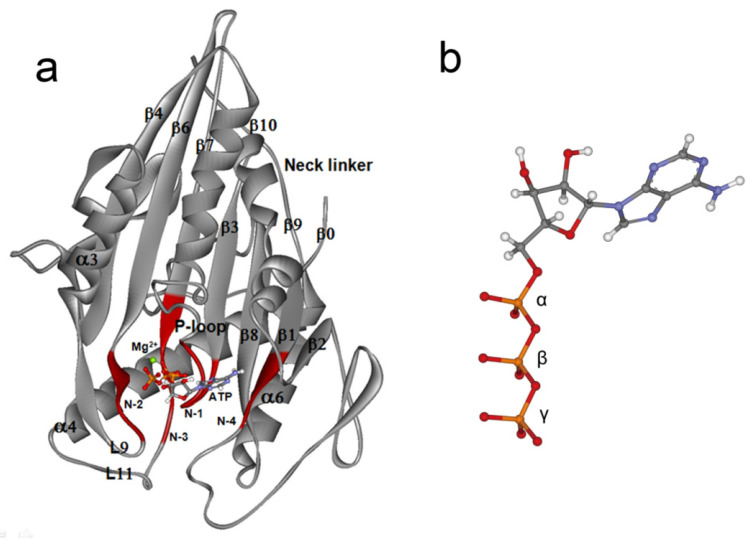
Structure of kinesin-1 nucleotide-binding pocket and ATP (adenosine triphosphate) molecule. (**a**) Kinesin-1 motor domain. The four motifs of kinesin-1 nucleotide-binding pocket are highlighted in red. The bound ATP molecule and Mg^2+^ are shown in the ball and stick mode. (**b**) Structure of ATP molecule. This figure was produced using Discovery studio 3.5 visualizer.

Kinesin-1 can catalyze ATP hydrolysis and use the energy stored in the ATP molecule to realize directional movement [22]. Since both the nucleotide-binding and the microtubule-binding sites of the kinesin-1 locate in the motor domain, the research on the conformational changes in different nucleotide-bound states of kinesin-1 is focused on the motor domain. The motor domain of kinesin-1 has a typical globular structure with a central β-sheet of eight strands in the center and three α-helices on either side of the central β-sheet [23,24,25]. The nucleotide-binding and the microtubule-binding sites of kinesin-1 locate on the opposite sides of the central β-sheet. Figure 2 shows the structures of the ATP molecule and the kinesin-1 nucleotide-binding pocket. Similar to other ATPase, the nucleotide-binding pocket of kinesin-1 consists of four motifs [26,27], which are referred to as the N-1 motif (also called P-loop or Walker A sequence [28,29], Gly-X-X-X-X-Gly-Lys-Thr/Ser), the N-2 motif (also called switch-I, Asn-X-X-Ser-Ser-Arg), the N-3 motif (also called switch-II, Asp-X-X-Gly-X-Glu) and the N-4 motif (Arg-X-Arg-Pro). Table 1 depicts the sequence alignment of the nucleotide-binding site among the kinesin family members. The four motifs are highly conserved in the kinesin family, especially the N-3 motif (the switch-II), which is absolutely conservative in the whole family. The entire structure of nucleotide-binding pocket of kinesin-1 is a perfect match for ATP molecules in both geometry and interaction (Figure 2). The N-1, N-2 and N-3 motifs form a pocket structure [30]. The three phosphate groups of the ATP molecule, which have four negative charges, bind tightly into this pocket and, together with Mg^2+^, form salt bridges, hydrogen bonds and coordination bonds with the surrounding residues [30,31,32]. N-4 and N-1 form a geometrically matched hydrophobic pocket for the adenosine ring of ATP. The hydrophobic stacking between the pocket and the adenosine ring of the ATP molecule contributes to the binding and recognition of nucleotide with the nucleotide-binding pocket of kinesin-1.

**Figure 3:**
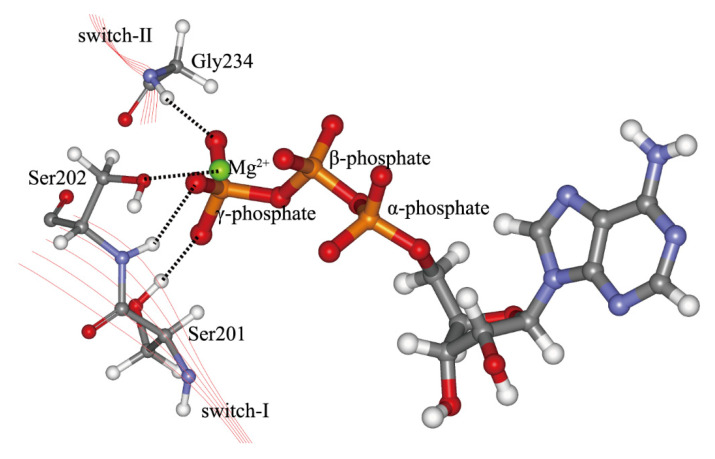
Interactions of Ser201, Ser202 on switch-I and Gly234 on switch-II with the γ-phosphate of the ATP molecule. The side chain of Ser202 interacts directly with Mg^2+^. This figure is produced based on the crystal structure 4HNA [61]. The residue numbering of structure 1MKJ [12] is used. This figure was produced by using Discovery studio 3.5 visualizer.

It has been proved in experiments that the key force-generation step of kinesin-1 in the walking process, i.e., the neck linker docking to the motor domain, is induced by the binding of ATP molecule to the nucleotide-binding pocket, not by the hydrolysis of ATP molecule [9]. The microsecond-timescale all-atomic molecular dynamics (MD) simulations by Hwang et al. [33] show that the conformational changes of α0, switch-I (N-2 motif of nucleotide-binding site) and L5 loop of kinesin-1 facilitate the binding of ATP molecule to the nucleotide-binding pocket. The entrance of the ATP molecule into the nucleotide-binding pocket can induce a series of conformational changes of the pocket, which can be amplified and finally transmitted to the neck linker portion to initiate the docking movement of neck linker to the motor domain [12,34,35,36,37,38,39,40,41,42]. The first step of this process is the sensing of the existence of the ATP molecule [43,44]. Analogous to the G protein [26,45], the serines on the N-2 motif (the conserved fourth and fifth residues on the N-2 motif, see Table 1) and the glycine on the N-3 motif (the conserved fifth residue on the N-3 motif, see Table 1) of kinesin-1 nucleotide-binding pocket are considered to sense the existence of ATP molecule through directly interacting with the γ-phosphate of ATP (Figure 3). Therefore, the switch-I and switch-II are the γ-phosphate sensors of kinesin-1. Both the motifs have large conformational changes upon ATP binding into the nucleotide-binding pocket of kinesin family [46]. Comparison of the structures of the motor domain in different nucleotide-binding states shows that the switch-I and switch-II are in an open conformation in the nucleotide-free state (apo state). After ATP entering the binding pocket, the switch-I and switch-II will cover the ATP molecule and change to the closed state.

To date and to our knowledge, the systematic research on the detailed mechanism of ATP hydrolysis catalyzed by kinesin-1 is still lacking. Parke et al. [32] investigated the ATP-hydrolysis process of Eg5 protein (belongs to the kinesin-5 subfamily) and proposed a two-water mechanism of ATP hydrolysis. Based on the structure obtained by Parke et al. [32], McGrath et al. [47] gave more details about the ATP hydrolysis by using quantum-mechanical/molecular-mechanical (QM/MM) metadynamics. In this two-water model, two water molecules together with several key residues in the active site form a proton transfer network, which performs the nucleophilic attack of the γ-phosphate of the ATP molecule and finally causes the cleavage of the β-γ bridging bond of ATP. When the ATP molecule docks to the nucleotide-binding pocket, Arg234 (amino acid sequence of PDB structure 3HQD [32], corresponding to Arg203 in 1MKJ [12]) on switch-I and Glu270 (Glu236 in 1MKJ) on switch-II can form a salt bridge. In this way, the switch-I and switch-II connect with each other and the nucleotide-binding pocket is closed. Closure of the nucleotide-binding pocket after ATP binding can protect the interactions between ATP and the related residues from the influence of the surrounding water molecules and ensure the environment for ATP hydrolysis [32]. Considering the highly conserved nucleotide-binding pocket of kinesin family, the ATP-hydrolysis mechanism proposed by Parke et al. and McGrath et al. should at least partly apply to the ATP-hydrolysis process of kinesin-1.

From the above discussions, the energy of ATP molecule is used by kinesin-1 in two different forms. First, the binding of charged ATP and Mg^2+^ to kinesin-1 motor domain can lower the energy of the system and produce a series of conformational changes of kinesin-1 nucleotide-binding pocket [9]. These conformational changes can be transmitted and amplified by other elements and initiate the force-generation process of kinesin-1. After this step, kinesin-1 catalyzes the ATP-hydrolysis process. The ATP hydrolysis is an exoergic process. The energy released from the ATP hydrolysis gives the product Pi a high energy and escapes from the constraint of the surrounding residues of the nucleotide-binding pocket. This process will induce another series of the conformational changes of the nucleotide-binding pocket that assists the motor domain to recover its initial conformation before ATP binding and prepare for the next mechanochemical cycle [49]. 

## 3. Motor-Domain Rotation Induced by ATP Binding Initiates the Force-Generation Process of Kinesin-1

**Figure 4:**
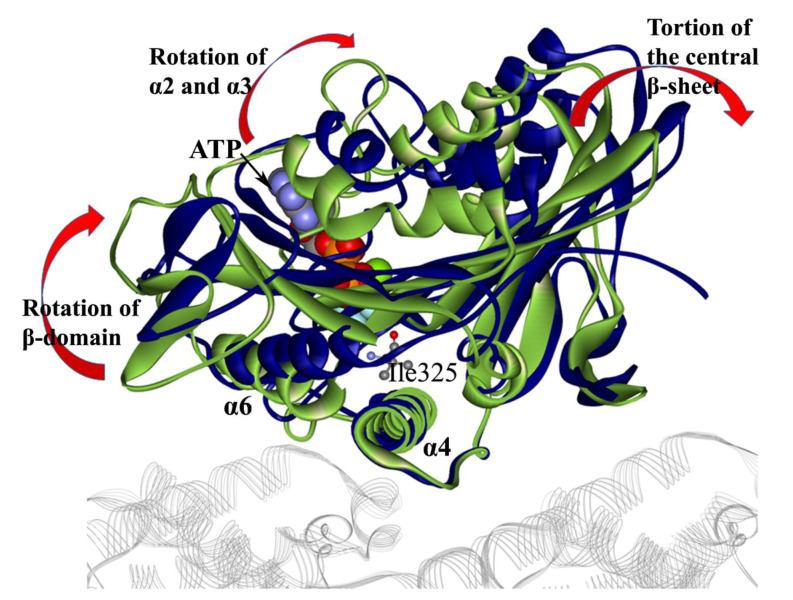
Superposition of the X-ray crystal structures of kinesin-1 and tubulin complex in nucleotide-free (colored in green, PDB ID: 4LNU [62]) and ATP-bound state (colored in blue, PDB ID: 4HNA [61]). The tubulins (represented in the gray line ribbon mode) and α4 helices of the two structures coincide in the superimposed structure. The nucleotide-binding side of the central β-sheet shows a large rotation due to the ATP binding. The microtubule-binding side is relatively stable except for α6, which has a large conformational change. The ATP molecule and the Ile325 (buried in the “docking cleft” when the neck linker is in the docked state) are explicitly shown to depict the position of the ATP-binding site and the “docking cleft” of kinesin-1. This figure was produced by using Discovery studio 3.5 visualizer.

A key question of kinesin-1 working mechanism is how kinesin-1 transforms the chemical energy stored in the ATP molecule to the mechanical energy for its movement along the microtubule [60]. The answer to this question starts from the study of the effect of ATP binding to kinesin-1. The conformational changes induced by ATP binding can be identified through the comparison of the conformations before and after ATP binding. The N-1 motif (the P-loop) is treated as the ‘finger print’ of the nucleotide-binding site of ATPase. When comparing the motor-domain structures in different nucleotide-bound states, it is used to keep the N-1 motifs of these structures coincided [27,30]. Taking the lever-arm motion of myosin in the different nucleotide-bound states as an analogy, Vale et al. [10] proposed that the α4 helix (also called switch-II helix) of kinesin-1 is similar to the “relay helix” of myosin. The piston-like motion of the α4 helix in different nucleotide-binding states could realize the long-range communication between the nucleotide-binding pocket and the neck linker. However, the cryo-electron microscope (cryo-EM) and X-ray structures of the kinesin–tubulin complex indicate that α4 helix is the main microtubule-binding site of kinesin-1 [12,27,39,61,62]. Comparison of structures of kinesin-1 in the nucleotide-free and ATP-bound state confirms that in the process of ATP entering the nucleotide-binding pocket, the α4 helix is fixed on the microtubule (Figure 4) [38,63,64,65].

**Figure 5:**
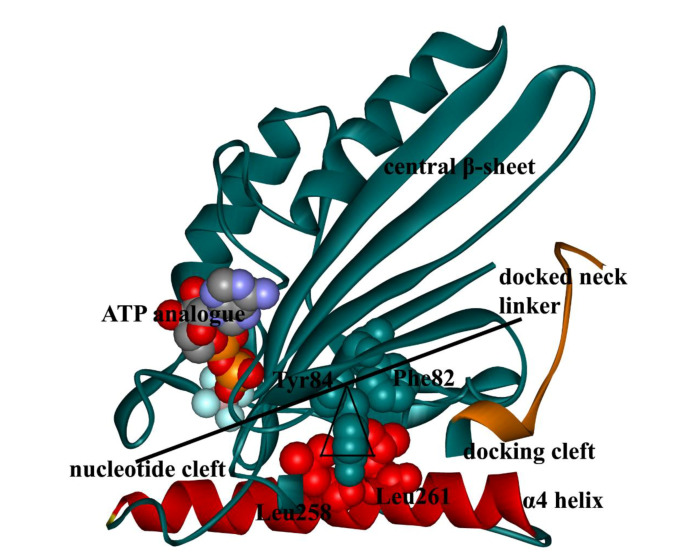
Diagram of the “seesaw” model (PDB ID: 4HNA [61]). The “fulcrum” is composed of Phe82, Tyr84, Leu258 and Leu261. The α4 helix (red) provides the support for the “seesaw”. The 4HNA structure is in the ATP-bound state, with an ATP analogue (ADP-AlF_4_^-^) in the nucleotide-binding pocket. In this state, the nucleotide cleft is in the closed state and the docking cleft is in the open state. This figure was produced by using Discovery studio 3.5 visualizer.

The superimposed structure of the kinesin–tubulin complex in kinesin-1 nucleotide-free and ATP-bound states shows that the ATP-binding process induces a rotation of the central β-sheet relative to the fixed α4 helix (Figure 4). Sindelar et al. [12,37,38,39] proposed a “seesaw” model to describe the mechanical transition from the nucleotide-binding pocket to the neck linker. In this model, the interactions between Asn255 (the amino acid sequence of PDB structure 1MKJ [12]) of the motor domain and tubulin stabilize the N-terminal part of α4 helix. The whole motor domain is analogous to a seesaw with the central β-sheet as the board (Figure 5). The fulcrum of the seesaw consists of Leu258, Leu261 on α4 and Phe82, Tyr84 on the central β-sheet. Tilting of the central β-sheet controls the opening of the nucleotide-binding pocket and the neck linker docking pocket located at the two ends of the “seesaw”, respectively. The binding of the ATP molecule to the nucleotide-binding pocket pulls one end of the “seesaw” and induces the tilting of the other end, which opens the neck linker docking pocket. The entrance of Ile325 into the neck linker docking pocket finally triggers the docking movement of neck linker to the motor domain.

With the recent progress of experimental methods, the high-resolution cryo-EM structures and X-ray crystal structures of the kinesin–tubulin complex in different nucleotide-bound states have been obtained [61,62,66,67,68,69]. These structures provide much more information about the conformational changes induced by the binding of the ATP molecule into the nucleotide-binding pocket. Based on these structures, experimental [66,67,70,71,72] and theoretical [33,73,74] research confirms that the whole motor domain can be divided into three subdomains. The conformational changes induced by ATP binding can be more exquisitely described as the conformational changes and relative motion of the subdomains. The “alternating cleft” model is proposed on the basis of the above results [67,72]. In this model, there are several clefts on the motor domain in different nucleotide-bound states. In the nucleotide-free state, there is a nucleotide cleft between P-loop (N-1 motif) and switch-II (N-3 motif). The ATP molecule can enter the binding pocket through this cleft and consequently pull the P-loop ~4 Å toward the microtubule. Interactions between P-loop and switch-II in this state can close the nucleotide cleft and induce the rotation of the α2 helix and the central β-sheet to open another cleft, i.e., the “docking cleft”, for the neck linker. Opening of the docking cleft permits the docking movement of the neck linker to the motor domain. In the process of nucleotide-cleft closing and docking-cleft opening, the central β-sheet has torsion and twisting (Figure 4 and Figure 5) [33,67,74]. Distortion of the central β-sheet was analogous to an archery bow by Shang et al. [67]. In the nucleotide-free state, the P-loop and switch-II stay apart and the “bow” is in the relaxed state. After ATP binding, interactions between P-loop and switch-II result in the distortion of the central β-sheet and initiate the neck linker docking process. The amphipathic nature of the α4 helix facilitates this subdomain movement [75]. Besides the kinesin-1 subfamily, the rotation of the central β-sheet of Kif1A (belonging to the kinesin-3 subfamily) was investigated using structure-based coarse-grained simulations [76] and showed similar conformational changes to that of kinesin-1.

## 4. Docking Movement of Kinesin-1 Neck Linker to Motor Domain

**Figure 6:**
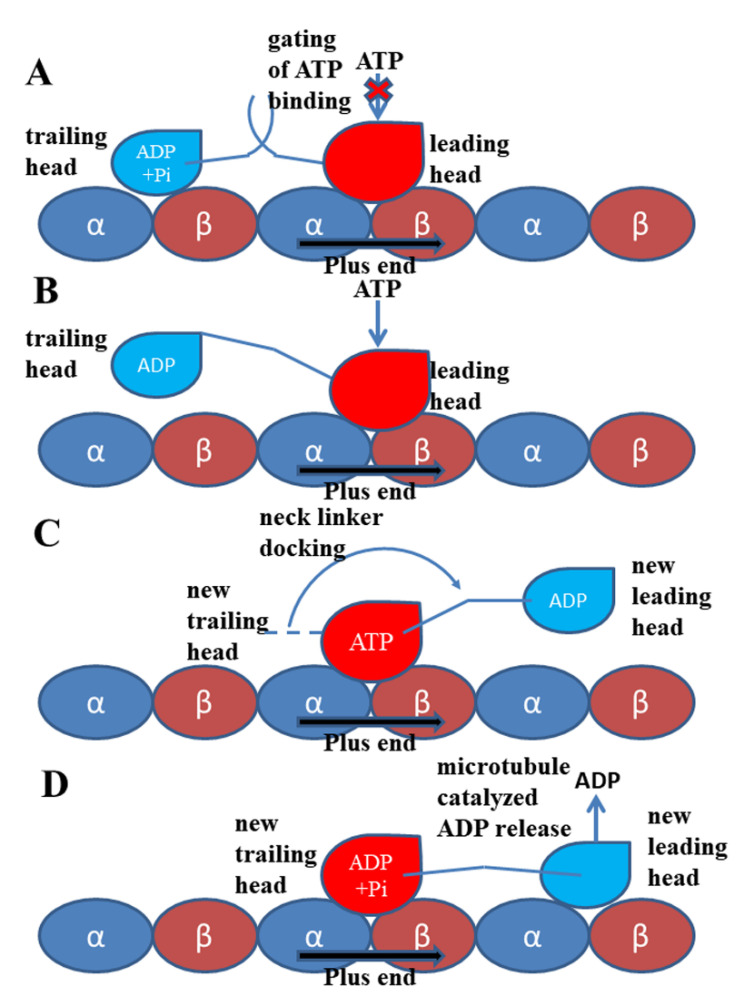
Walking and mechanochemical coupling mechanisms of kinesin-1. (**A**) Both motor domains bind strongly to the microtubule (the same as Figure 1). In this state, the internal strain between the two motor domains and the backward-orientated neck linker of the leading head inhibit the binding of the ATP molecule to this motor domain. (**B**) Detachment of the trailing head from the microtubule releases the internal strain and the restriction to the orientation of the neck linker of the leading head. The ATP molecule can bind to the leading head. (**C**) The neck linker docking induced by the ATP binding pulls the trailing head to the next binding site on the microtubule. (**D**) The ADP-bound state new leading head binds to the microtubule. The ADP molecule releases from this head quickly due to the catalysis of the microtubule.

In 1999, Rice et al. [9] proved that the conformational change of the neck linker region is a key step in the “walking” process of kinesin-1. Subsequent experimental and theoretical research [10,12,35,77,78,79], reviewed in [80], show that the conformational change of the neck linker from the undocked state to the docked state can pull the other head to its next binding site on the microtubule (the relative position of the two motor domains is shown in Figure 1 and the walking pattern of kinesin-1 is shown in Figure 6). In this way, kinesin-1 “walks” one step on microtubule lattice (the hand-over-hand manner) [81,82,83,84,85,86]. The subdomain movements discussed in the above section can be transmitted to the neck linker and initiate the neck linker docking process.

The neck linker of kinesin-1 consists of 14 residues and can be divided into three parts according to their different conformational changes in the docking movement. From the N-terminal end of the neck linker, the first part is three residues (Lys323, Thr324 and Ile325, amino acid sequence of 1MKJ [12]), which connect to the α6 helix of the motor domain. After these three residues is the β9 portion of the neck linker. In the docked state of the neck linker, the β9 can form a β-sheet with the motor domain. The β9 consists of six residues, Lys326, Asn327, Thr328, Val329, Cys330 and Val331. The C-terminal end of the neck linker is the β10 portion. Five residues, Asn332, Val333, Glu334, Leu335 and Thr336, constitute the β10, which, similar to β9, forms a β-sheet with the motor domain in the docked state. The docking of the neck linker to the motor domain is realized through interactions of these residues with the motor domain [87]. However, the docking mechanisms of these three parts are different.

**Figure 7:**
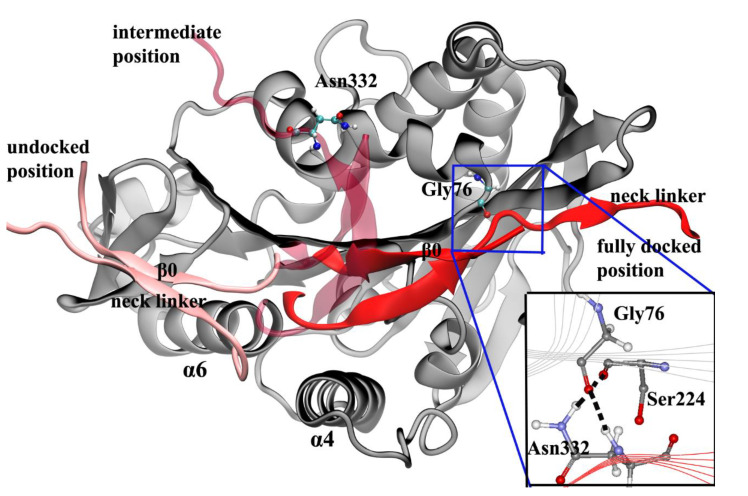
Diagram of the neck linker docking process. The neck linker in the initial (pink), intermediate (light red), and fully docked positions (red) are shown. In the intermediate position, the distance between the Asn332 and the Gly76 is beyond the force range of a hydrogen bond. The inset is the Asn latch structure in the fully docked state of the neck linker. This figure was produced using VMD (version 1.9.3) [99] and the inset was produced using Discovery studio 3.5 visualizer.

The first step of the neck linker docking is the large conformational change of the three residues on the N-terminal end. In the docked conformation of the neck linker, the three residues (Lys323, Thr324 and Ile325) form a half turn of α-helix structure along the C-terminal end of the α6 helix. This structure formed by the three residues is called the “extra turn” [88,89]. Formation of the extra turn is induced by the rotation of the central β-sheet. This step initiates the subsequent docking of the β9 to the motor domain. The main driving force of β9 docking movement comes from the bending of a special structure formed by β0 and β9, i.e., the “cover-neck bundle” (CNB) [90]. The function of the CNB structure was firstly proposed by Hwang et al. [90]. In the MD simulations of Hwang et al. [90], the CNB structure has the spontaneous tendency of bending to the motor domain. This result was also proved by subsequent experimental research [91]. It is worthy to note that this CNB mechanism is shared by Eg5 protein, which belongs to the kinesin-5 subfamily [41,92,93]. The CNB-induced neck linker docking may be a universal mechanism applied to the majority of the kinesins. To date, the mechanism of the third step of the neck linker docking, the docking of the β10 to the motor domain, is still unclear. Hwang et al. [90] proposed that the key event of the β10 docking should be the formation of a backbone hydrogen bond between Asn332 and Gly76. They call this hydrogen bond the Asn latch (Figure 7). In the steered molecular dynamics (SMD) simulations of Geng et al. [87], the Asn latch portion had a high strength. The high strength of this part originates from the cooperation of the hydrophobic residues around the Asn latch, which effectively protect the backbone hydrogen bond of the Asn latch [94]. Unfortunately, the formation mechanism of this Asn latch is still unclear. After the docking of β9, the distance between Asn332 and Gly76 is still far beyond the effective force range of a hydrogen bond (Figure 7). We speculate that, at this moment, the trailing head has detached from the microtubule since the motor-domain rotation of the leading head is sufficient to detach the trailing head [42]. The extra-turn formation and the β9 docking movement can pull the trailing head to the region near the next binding site on the microtubule. Interactions between the trailing head and microtubule or between the two heads [95] could pull the β10 to the plus end of the microtubule and facilitate the formation of the Asn latch. Consistent with this hypothesis, recent experimental results showed that the full neck linker docking process is completed upon ATP hydrolysis, not ATP binding [96,97,98]. There may be two factors affecting the formation of the Asn latch and the subsequent β10 docking. One is the binding of the trailing head to the next binding site on the microtubule and another is the conformational changes of the motor domain upon the ATP hydrolysis. However, we still cannot confirm which one is the dominant factor.

Geng et al. [88,89] proposed another function of the CNB structure, which participates in the initiation of the neck linker docking. In the MD simulations of Geng et al. [88], they constructed a model to represent the structure of the motor domain at the beginning of the neck linker docking (the undocked position of the neck linker in Figure 7). Using this structure as the starting point, Geng et al. realized the neck linker docking process in the MD simulation. According to their simulation trajectories, formation of the extra-turn structure is driven by the rotation of the central β-sheet. The mechanical pathway from the central β-sheet to the extra turn is through an initial CNB structure. However, the simulations of Geng et al. cannot show the complete rotational movement of the central β-sheet around α4 helix. An artificial force is exerted on the central β-sheet to mimic its rotation effect in their simulations. The results will be more persuasive if the complete process from ATP binding to neck linker docking is realized in the MD simulations instead of adding an artificial force.

## 5. Coupling Regulation of Kinesin-1 Mechanochemical Cycle

Kinesin-1 can walk unidirectionally and processively along the microtubule lattice. The backward step rarely happens for kinesin-1 in the normal condition, and only one ATP molecule is consumed to walk one step. These features in the walking process of kinesin-1 require that the chemical cycle and the mechanical cycle of the motor domain are highly coupled and regulated [100,101,102,103,104,105]. Since the conformations of the motor domain vary with the different nucleotide-bound states, the binding strength of the motor domain of kinesin-1 with the microtubule is different. The motor domain in the ADP-bound state binds weakly to the microtubule lattice and, in the other nucleotide-bound states, the motor domain binds to the microtubule strongly. The chemical-cycle regulation of kinesin-1 mainly has two aspects.

When kinesin-1 dissociates from the microtubule lattice, the two motor domains are in the ADP-bound state. The interactions between the motor domain and the tail domain effectively inhibit the release of an ADP molecule from the nucleotide-binding site [106,107,108,109,110]. In the walking process of kinesin-1, the spontaneous release of the ADP molecule is also very slow (~0.005–0.009 s^−1^ [111,112]) though the inhibition effect of the tail domain is absent. One of the mechanochemical regulations comes from the walking track of kinesin-1, i.e., the microtubule lattice [113]. Binding of the motor domain in the ADP-bound state with the microtubule accelerates the ADP-dissociation rate of this motor domain (~9 s^−1^ [111,112], increasing ~1000 fold compared with the spontaneous release rate). The microtubule-catalyzed ADP release can rapidly turn the motor domain into the nucleotide-free state to wait for the entrance of a new ATP molecule. The information-transition pathway from microtubule binding to the release of ADP molecule is still unclear. Shang et al. [67] found that the Asn255 of motor domain (the “linchpin”) plays a key role in the motor domain’s microtubule-response pathway. The MD simulations of Jin et al. [65] proposed that, besides Asn255, Lys237 on the switch-II has interactions with a charged group consisting of Glu414, Glu417 and Glu420 on the α-tubulin. This direct contact between the nucleotide-binding pocket and microtubule may also participate in sensing the existence of the tubulin. The nucleotide-bound state of kinesin directly influences the binding strength of the motor domain with a microtubule. The ADP release catalyzed by the microtubule ensures that the motor domain converts to the nucleotide-free state rapidly and then binds strongly with the microtubule lattice. In this way, the rapid and processive movement of kinesin-1 along the microtubule is guaranteed.

The second regulation of the kinesin-1 mechanochemical cycle is the gating of ATP binding to the nucleotide-binding pocket. The ATP molecule can bind with the motor domain in the nucleotide-free state. However, to avoid the futile hydrolysis of ATP, the ATP molecule can only enter the binding pocket and be hydrolyzed under specific conditions. As discussed in the above section, the binding of ATP into the binding pocket of the motor domain can induce a series of conformational changes of the motor domain and finally trigger the neck linker docking movement. The neck linker docking of one motor domain can pull the other motor domain forward to the next binding site on the microtubule [85,86]. The motor domain, which is pulled forward, becomes the new leading head and the other one becomes the new trailing head (Figure 6). The ADP molecule rapidly releases from the new leading head due to the catalyzed effect of the microtubule that converts the motor domain into the nucleotide-free state. At this moment, both motor domains of the kinesin-1 dimer bind to the microtubule with the leading head in the nucleotide-free state (strong microtubule-bound state) and trailing head in the ATP/ADP·Pi-bound state (strong microtubule-bound state) (Figure 1 and Figure 6) [72]. This state in the walking process of kinesin-1 is called the “ATP-waiting state” [114]. Experiments had confirmed that the coiled coil keeps in the coiled conformation in the walking cycle of kinesin-1 [115]. Because the length of the neck linkers of the two motor domains is approximately equal to the distance between the two kinesin-binding sites on the microtubule (~8 nm), in the ATP-waiting state, there is an internal strain between the two motor domains through the neck linkers [116,117,118,119,120,121,122,123]. Due to the existence of the internal strain, the ATP molecule cannot bind stably with the motor domain and the hydrolysis of ATP will not occur [124]. Subsequent experiments [121,125] proposed that the direction of the neck linker plays a key role in the regulation of the ATP binding rather than the internal strain. As a matter of fact, these two factors are not contradictory. When the internal strain exists, the neck linker of the leading head naturally points to the minus direction of the microtubule, which is considered to prevent the ATP binding. When the ATP hydrolysis and the Pi release complete, the trailing head gets into the ADP-bound state. In this state, the trailing head will bind with the microtubule weakly or detach from the microtubule [64,95,126,127] and the internal strain between the two motor domains disappears. With the release of the restriction from the internal strain, the neck linker of the leading head cannot maintain the backward orientation and, thus, the inhibition to ATP binding is relieved. Only in this way can the ATP molecule bind with the nucleotide-free motor domain and the next mechanochemical cycle start. Therefore, the gating of ATP binding is another regulation of the kinesin-1 chemical cycle. Without the regulation of the ATP-binding process, the leading head in the nucleotide-free state would bind and hydrolyze the ATP molecule when the trailing head is still in the strong microtubule-bound state and a futile ATP hydrolysis cycle would occur.

A recent review by Hancock [105] discussed in detail the relationship between the gating and the processivity of kinesin-1. In our view, the disruption of the processivity is due to the kinesin-1 binding with the microtubule weakly, in which state the motor domains bound with microtubule are all in ADP-bound states. Since the disruption of the above two gating processes may lead to this state, they would raise the possibility of the detachment of kinesin-1 from the microtubule. However, the impact of the impaired gating on the processivity of kinesin-1 is determined by the possibility of the appearance of the weak microtubule-bound state, not directly by the introduced inhibition of the gating.

The mechanochemical coupling of kinesin-1 is realized through the regulation of the ATP-binding and ADP-dissociation processes. It is worthwhile to note that the regulation mechanisms of kinesin subfamilies are different [128]. For example, though the motor domains are similar, the mitotic centromere-associated kinesin (MCAK, a typical kinesin-13 member) shows a large difference in the mechanochemical regulation with kinesin-1. As described above, the motor domain of dissociated kinesin-1 is in the ADP-bound state and ADP dissociation is the rate-limiting step of kinesin-1 [112]. In contrast, the motor domain of dissociated MCAK is in the ATP-bound state and accordingly ATP hydrolysis is the rate-limiting step of MCAK [129]. The ATP hydrolysis of MCAK requires the binding of the motor domain with the microtubule and is a tubulin-catalyzed process. The MCAK depolymerizes the microtubule end. Thus, the ADP molecule will not be catalyzed to dissociate from the motor domain by microtubule lattice unless the motor domain arrives at the microtubule end. This is also different with the regulation of ADP release of kinesin-1.

## 6. Conclusions

After ~35 years of investigation, the walking mechanism and the mechanochemical coupling of kinesin-1 is much clearer now. The energy for mechanical walking of kinesin-1 comes from ATP. Because the ATP molecule is charged, the binding of ATP molecule into the nucleotide-binding site on the motor domain of kinesin-1 can induce a series of conformational changes in the motor domain. These conformational changes are finally transmitted to the neck linker and, thus, trigger the docking movement of the neck linker. In this paper, we try to combine the research results on the conformational changes and the mechanical pathway of this process to give a detailed description at the residue level. However, there are still some problems unresolved. For example, how is the β10 docking accomplished in the neck linker docking process? Most of the attractive features of kinesin-1 (e.g., unidirectional and processive movement on the microtubule lattice; one ATP molecule one step) need tight coupling between the mechanical cycle and the chemical cycle of kinesin-1. We discussed the regulations of two chemical processes, the ATP binding and the ADP release. However, detailed regulation mechanisms of these two processes are still unclear to date.

The discussions of this paper are restricted to the conformational changes of only one kinesin-1 protein. In vivo, the kinesin-1 proteins often interact with some microtubule-associated proteins or other proteins to work effectively [130]. To transport large organelles along the microtubules, the kinesin-1 proteins often cooperate to achieve a high moving speed. Besides the regulation of the ADP release of kinesin-1 by the microtubule, the post-translational modifications of the microtubules can also affect the activity of the kinesin-1 [131,132,133]. The detailed mechanisms of all these aspects at the residue level are still unknown and we still have a long way to go to achieve a complete understanding of kinesin mechanism.

## Figures and Tables

**Table 1:** Sequence alignment of the nucleotide-binding site of the kinesin family.

Kinesin Family ^1^	N-1	N-2	N-3	N-4
Kinesin-1 ^2^	G Q T S S G K T H	N E H S S R	D L A G S E	R F R P
Kinesin-2 ^3^	G Q T G A G K T Y	N D T S S R	D L A G S E	R C R P
Kinesin-3 ^4^	G Q T G S G K S Y	N D T S S R	D L A G S E	R V R A
Kinesin-4 ^5^	G Q T G S G K T Y	N S Q S S R	D L A G S E	R C R P
Kinesin-5 ^6^	G Q T G T G K T F	N A Y S S R	D L A G S E	R C R P
Kinesin-6 ^7^	G V T N S G K T Y	N Q Q S S R	D L A G S E	R I R P
Kinesin-7 ^8^	G Q T A S G K T Y	N Q R S S R	D L A G S E	R V R P
Kinesin-8 ^9^	G P T G C G K T Y	N Q T S S R	D L A G S E	R V R P
Kinesin-9 ^10^	G Q T G A G K T Y	N K N S S R	D L A G S E	R V K P
Kinesin-10 ^11^	G Q T G T G K S Y	N S N S S R	D L A G S E	R E A P
Kinesin-12 ^12^	G Q T G S G K T F	N R E S S R	D L A G S E	R I R P
Kinesin-13 ^13^	G Q T G S G K T H	N S N S S R	D L A G S E	R K R P
Kinesin-14 ^14^	G Q T G S G K T Y	N E R S S R	D L A G S E	R I R P

^1^ All the sequences in this table are taken from a representative structure of the corresponding subfamily. The footnotes denote the structures used (PDB ID). The conserved sites are highlighted in red color. ^2^ 1MKJ [12]; ^3^ 2VVG [48]; ^4^ 2OWM [49]; ^5^ 3ZFD [50]; ^6^ 1II6 [51]; ^7^ 5ND2 [52]; ^8^ 1T5C [53]; ^9^ 5GSZ [54]; ^10^ 3NWN [55]; ^11^ 3DC4 [56]; ^12^ 4BN2 [57]; ^13^ 5MIO [58]; ^14^ 2NCD [59].

## Abbreviations

ATP	adenosine triphosphate
ADP	adenosine diphosphate
MD	molecular dynamics
SMD	steered molecular dynamics
QM/MM	quantum-mechanical/molecular-mechanical
PDB	protein data bank
EM	electron microscope
CNB	cover-neck bundle
MCAK	mitotic centromere-associated kinesin

## References

[1] Lawrence C.J., Kelly Dawe R., Christie K.R., Cleveland D.W., Dawson S.C., Endow S.A., Goldstein L.S.B., Goodson H.V., Hirokawa N., Howard J. (2004). A standardized kinesin nomenclature. J. Cell Biol..

[2] Vale R.D. (2003). The molecular motor toolbox for intracellular transport. Cell.

[3] Hirokawa N., Noda Y., Tanaka Y., Niwa S. (2009). Kinesin superfamily motor proteins and intracellular transport. Nat. Rev. Mol. Cell Biol..

[4] Hirokawa N., Tanaka Y. (2015). Kinesin superfamily proteins (KIFs): Various functions and their relevance for important phenomena in life and diseases. Exp. Cell Res..

[5] Brady S.T. (1985). A novel brain ATPase with properties expected for the fast axonal transport motor. Nature.

[6] Vale R.D., Reese T.S., Sheetz M.P. (1985). Identification of a novel force-generating protein, kinesin, involved in microtubule-based motility. Cell.

[7] Guo S.K., Shi X.X., Wang P.Y., Xie P. (2019). Run length distribution of dimerized kinesin-3 molecular motors: Comparison with dimeric kinesin-1. Sci. Rep. UK.

[8] Cockburn J.J.B., Hesketh S.J., Mulhair P., Thomsen M., O’Connell M.J., Way M. (2018). Insights into kinesin-1 activation from the crystal structure of KLC2 bound to JIP3. Structure.

[9] Rice S., Lin A.W., Safer D., Hart C.L., Naber N., Carragher B.O., Cain S.M., Pechatnikova E., Wilson-Kubalek E.M., Whittaker M. (1999). A structural change in the kinesin motor protein that drives motility. Nature.

[10] Vale R.D., Milligan R.A. (2000). The way things move: Looking under the hood of molecular motor proteins. Science.

[11] Case R.B., Rice S., Hart C.L., Ly B., Vale R.D. (2000). Role of the kinesin neck linker and catalytic core in microtubule-based motility. Curr. Biol..

[12] Sindelar C.V., Budny M.J., Rice S., Naber N., Fletterick R., Cooke R. (2002). Two conformations in the human kinesin power stroke defined by X-ray crystallography and EPR spectroscopy. Nat. Struct. Biol..

[13] Tomishige M., Stuurman N., Vale R.D. (2006). Single-molecule observations of neck linker conformational changes in the kinesin motor protein. Nat. Struct. Mol. Biol..

[14] Singh S.K., Pandey H., Al-Bassam J., Gheber L. (2018). Bidirectional motility of kinesin-5 motor proteins: Structural determinants, cumulative functions and physiological roles. Cell. Mol. Life Sci..

[15] Yamagishi M., Shigematsu H., Yokoyama T., Kikkawa M., Sugawa M., Aoki M., Shirouzu M., Yajima J., Nitta R. (2016). Structural basis of backwards motion in kinesin-1-kinesin-14 chimera: Implication for kinesin-14 motility. Structure.

[16] Desai A., Verma S., Mitchison T.J., Walczak C.E. (1999). Kin I kinesins are microtubule-destabilizing enzymes. Cell.

[17] Hunter A.W., Caplow M., Coy D.L., Hancock W.O., Diez S., Wordeman L., Howard J. (2003). The kinesin-related protein MCAK is a microtubule depolymerase that forms an ATP-hydrolyzing complex at microtubule ends. Mol. Cell.

[18] Helenius J., Brouhard G., Kalaidzidis Y., Diez S., Howard J. (2006). The depolymerizing kinesin MCAK uses lattice diffusion to rapidly target microtubule ends. Nature.

[19] Walczak C.E., Gayek S., Ohi R. (2013). Microtubule-depolymerizing kinesins. Annu. Rev. Cell Dev. Biol..

[20] Schnitzer M.J., Block S.M. (1997). Kinesin hydrolyses one ATP per 8-nm step. Nature.

[21] Coy D.L., Wagenbach M., Howard J. (1999). Kinesin takes one 8-nm step for each ATP that it hydrolyzes. J. Biol. Chem..

[22] Cochran J.C. (2015). Kinesin motor enzymology: Chemistry, structure, and physics of nanoscale molecular machines. Biophys. Rev..

[23] Sack S., Müller J., Marx A., Thormählen M., Mandelkow E.-M., Brady S.T., Mandelkow E. (1997). X-ray structure of motor and neck domains from rat brain kinesin. Biochemistry.

[24] Kozielski F., Sack S., Marx A., Thormählen M., Schönbrunn E., Biou V., Thompson A., Mandelkow E.-M., Mandelkow E. (1997). The crystal structure of dimeric kinesin and implications for microtubule-dependent motility. Cell.

[25] Kull F.J., Sablin E.P., Lau R., Fletterick R.J., Vale R.D. (1996). Crystal structure of the kinesin motor domain reveals a structural similarity to myosin. Nature.

[26] Sablin E.P., Kull F.J., Cooke R., Vale R.D., Fletterick R.J. (1996). Crystal structure of the motor domain of the kinesin-related motor ncd. Nature.

[27] Smith C.A., Rayment I. (1996). Active site comparisons highlight structural similarities between myosin and other P-loop proteins. Biophys. J..

[28] Walker J.E., Saraste M., Runswick M.J., Gay N.J. (1982). Distantly related sequences in the α- and β-subunits of ATP synthase, myosin, kinases, and other ATP-requiring enzymes and a common nucleotide binding fold. EMBO J..

[29] Saraste M., Sibbald P.R., Wittinghofer A. (1990). The P-loop-a common motif in ATP- and GTP-binding proteins. Trends Biol. Sci..

[30] Sack S., Kull F.J., Mandelkow E. (1999). Motor proteins of the kinesin family-Structures, variations, and nucleotide binding sites. Eur. J. Biochem..

[31] Kull F.J., Endow S.A. (2002). Kinesin: Switch I & II and the motor mechanism. J. Cell Sci..

[32] Parke C.L., Wojcik E.J., Kim S.Y., Worthylake D.K. (2010). ATP hydrolysis in Eg5 kinesin involves a catalytic two-water mechanism. J. Biol. Chem..

[33] Hwang W., Lang M.J., Karplus M. (2017). Kinesin motility is driven by subdomain dynamics. eLife.

[34] Vale R.D. (1996). Switches, latches, and amplifiers: Common themes of G proteins and molecular motors. J. Cell Biol..

[35] Skiniotis G., Surrey T., Altmann S., Gross H., Song Y.H., Mandelkow E., Hoenger A. (2003). Nucleotide-induced conformations in the neck region of dimeric kinesin. EMBO J..

[36] Skiniotis G., Cochran J.C., Müller J., Mandelkow E., Gilbert S.P., Hoenger A. (2004). Modulation of kinesin binding by the C-termini of tubulin. EMBO J..

[37] Sindelar C.V., Downing K.H. (2007). The beginning of kinesin’s force-generating cycle visualized at 9-Å resolution. J. Cell Biol..

[38] Sindelar C.V., Downing K.H. (2010). An atomic-level mechanism for activation of the kinesin molecular motors. Proc. Natl. Acad. Sci. USA.

[39] Sindelar C.V. (2011). A seesaw model for intermolecular gating in the kinesin motor protein. Biophys. Rev..

[40] Kikkawa M., Hirokawa N. (2006). High-resolution cryo-EM maps show the nucleotide binding pocket of KIF1A in open and closed conformations. EMBO J..

[41] Goulet A., Behnke-Parks W.M., Sindelar C.V., Major J., Rosenfeld S.S., Moores C.A. (2012). The structural basis of force generation by the mitotic motor Kinesin-5. J. Biol. Chem..

[42] Geng Y., Liu S., Ji Q., Yan S. (2014). Mechanical amplification mechanism of kinesin’s beta-domain. Arch. Biochem. Biophys..

[43] Kikkawa M., Sablin E.P., Okada Y., Yajima H., Fletterick R.J., Hirokawa N. (2001). Switch-based mechanism of kinesin motors. Nature.

[44] Sablin E.P., Fletterick R.J. (2001). Nucleotide switches in molecular motors: Structural analysis of kinesins and myosins. Curr. Opin. Struct. Biol..

[45] Fisher A.J., Smith C.A., Thoden J.B., Smith R., Sutoh K., Holden H.M., Rayment I. (1995). X-ray structures of the myosin motor domain of dictyostelium discoideum complexed with MgADP·BeF_x_ and MgADP·AlF_4_^−^. Biochemistry.

[46] Minehardt T.J., Cooke R., Pate E., Kollman P.A. (2001). Molecular dynamics study of the energetic, mechanistic, and structural implications of a closed phosphate tube in ncd. Biophys. J..

[47] McGrath M.J., Kuo I.F., Hayashi S., Takada S. (2013). Adenosine triphosphate hydrolysis mechanism in kinesin studied by combined quantum-mechanical/molecular-mechanical metadynamics simulations. J. Am. Chem. Soc..

[48] Hoeng J.C., Dawson S.C., House S.A., Sagolla M.S., Pham J.K., Mancuso J.J., Löwe J., Cande W.Z. (2008). High-resolution crystal structure and in vivo function of a kinesin-2 homologue in Giardia intestinalis. Mol. Biol. Cell.

[49] Nitta R., Okada Y., Hirokawa N. (2008). Structural model for strain-dependent microtubule activation of Mg-ADP release from kinesin. Nat. Struct. Mol. Biol..

[50] Chang Q., Nitta R., Inoue S., Hirokawa N. (2013). Structural basis for the ATP-induced isomerization of kinesin. J. Mol. Biol..

[51] Turner J., Anderson R., Guo J., Beraud C., Fletterick R., Sakowicz R. (2001). Crystal structure of the mitotic spindle kinesin Eg5 reveals a novel conformation of the neck-linker. J. Biol. Chem..

[52] Atherton J., Yu I.M., Cook A., Muretta J.M., Joseph A., Major J., Sourigues Y., Clause J., Topf M., Rosenfeld S.S. (2017). The divergent mitotic kinesin MKLP2 exhibits atypical structure and mechanochemistry. eLife.

[53] Garcia-Saez I., Yen T., Wade R.H., Kozielski F. (2004). Crystal structure of the motor domain of the human kinetochore protein CENP-E. J. Mol. Biol..

[54] Wang D.D., Nitta R., Morikawa M., Yajima H., Inoue S., Shigematsu H., Kikkawa M., Hirokawa N. (2016). Motility and microtubule depolymerization mechanisms of the Kinesin-8 motor, KIF19A. eLife.

[55] Zhu H., Tempel W., He H., Shen Y., Wang J., Brothers G., Landry R., Arrowsmith C.H., Edwards A.M., Sundstrom M. (2010). Crystal structure of the human KIF9 motor domain in complex with ADP. PDB.

[56] Cochran J.C., Sindelar C.V., Mulko N.K., Collins K.A., Kong S.E., Hawley R.S., Kull F.J. (2009). ATPase cycle of the nonmotile kinesin NOD allows microtubule end tracking and drives chromosome movement. Cell.

[57] Klejnot M., Falnikar A., Ulaganathan V., Cross R.A., Baas P.W., Kozielski F. (2014). The crystal structure and biochemical characterization of Kif15: A bifunctional molecular motor involved in bipolar spindle formation and neuronal development. Acta Crystallogr. D. Biol. Crystallogr..

[58] Wang W., Cantos-Fernandes S., Lv Y., Kuerban H., Ahmad S., Wang C., Gigant B. (2017). Insight into microtubule disassembly by kinesin-13s from the structure of Kif2C bound to tubulin. Nat. Commun..

[59] Sablin E.P., Case R.B., Dai S.C., Hart C.L., Ruby A., Vale R.D., Fletterick R.J. (1998). Direction determination in the minus-end-directed kinesin motor ncd. Nature.

[60] Block S.M. (2007). Kinesin motor mechanics: Binding, stepping, tracking, gating, and limping. Biophys. J..

[61] Gigant B., Wang W., Dreier B., Jiang Q., Pecqueur L., Plückthun A., Wang C., Knossow M. (2013). Structure of a kinesin-tubulin complex and implications for kinesin motility. Nat. Struct. Mol. Biol..

[62] Cao L., Wang W., Jiang Q., Wang C., Knossow M., Gigant B. (2014). The structure of apo-kinesin bound to tubulin links the nucleotide cycle to movement. Nat. Comm..

[63] Asenjo A.B., Krohn N., Sosa H. (2003). Configuration of the two kinesin motor domains during ATP hydrolysis. Nat. Struct. Biol..

[64] Asenjo A.B., Sosa H. (2009). A mobile kinesin-head intermediate during the ATP-waiting state. Proc. Natl. Acad. Sci. USA.

[65] Jin Y., Geng Y., Lü L., Ma Y., Lü G., Zhang H., Ji Q. (2017). Anchor effect of interactions between kinesin’s nucleotide-binding pocket and microtubule. Cel. Mol. Bioeng..

[66] Atherton J., Farabella I., Yu I., Rosenfeld S.S., Houdusse A., Topf M., Moores C.A. (2014). Conserved mechanisms of microtubule-stimulated ADP release, ATP binding, and force generation in transport kinesins. eLife.

[67] Shang Z., Zhou K., Xu C., Csencsits R., Cochran J.C., Sindelar C.V. (2014). High-resolution structures of kinesin on microtubules provide a basis for nucleotide-gated force-generation. eLife.

[68] Wang W., Cao L., Wang C., Gigant B., Knossow M. (2015). Kinesin, 30 years later: Recent insights from structural studies. Protein Sci..

[69] Morikawa M., Yajima H., Nitta R., Inoue S., Ogura T., Sato C., Hirokawa N. (2015). X-ray and cryo-EM structures reveal mutual conformational changes of Kinesin and GTP-state microtubules upon binding. EMBO J..

[70] Muretta J.M., Jun Y., Gross S.P., Major J., Thomas D.D., Rosenfeld S.S. (2015). The structural kinetics of switch-1 and the neck linker explain the functions of kinesin-1 and Eg5. Proc. Natl. Acad. Sci. USA.

[71] Cao L., Cantos-Fernandes S., Gigant B. (2017). The structural switch of nucleotide-free kinesin. Sci. Rep. UK.

[72] Liu D., Liu X., Shang Z., Sindelar C.V. (2017). Structural basis of cooperativity in kinesin revealed by 3D reconstruction of a two-head-bound state on microtubules. eLife.

[73] Scarabelli G., Grant B.J. (2013). Mapping the structural and dynamical features of kinesin motor domains. PLoS Comput. Biol..

[74] Krukau A., Knecht V., Lipowsky R. (2014). Allosteric control of kinesin’s motor domain by tubulin: A molecular dynamics study. Phys. Chem. Chem. Phys..

[75] Ma Y., Li T., Jin Y., Geng Y., Ji Q. (2019). Shaft function of kinesin-1’s α4 helix in the processive movement. Cell. Mol. Bioeng..

[76] Liu F., Ji Q., Wang H., Wang J. (2018). Mechanochemical model of the power stroke of the single-headed motor protein KIF1A. J. Phys. Chem. B.

[77] Rice S., Cui Y., Sindelar C., Naber N., Matuska M., Vale R., Cooke R. (2003). Thermodynamic properties of the kinesin neck-region docking to the catalytic core. Biophys. J..

[78] Asenjo A.B., Weinberg Y., Sasa H. (2006). Nucleotide binding and hydrolysis induces a disorder-order transition in the kinesin neck-linker region. Nat. Struct. Mol. Biol..

[79] Budaitis B.G., Jariwala S., Reinemann D.N., Schimeret K.I., Scarabelli G., Grant B.J., Sept D., Lang M.J., Verhey K.J. (2019). Neck linker docking is critical for Kinesin-1 force generation in cells but at a cost to motor speed and processivity. eLife.

[80] Hwang W., Karplus M. (2019). Structural basis for power stroke vs. Brownian ratchet mechanisms of motor proteins. Proc. Natl. Acad. Sci. USA.

[81] Asbury C.L., Fehr A.N., Block S.M. (2003). Kinesin moves by an asymmetric hand-over-hand mechanism. Science.

[82] Kaseda K., Higuchi H., Hirose K. (2003). Alternate fast and slow stepping of a heterodimeric kinesin molecule. Nat. Cell Biol..

[83] Yildiz A., Tomishige M., Vale R.D., Selvin P.R. (2004). Kinesin walks hand-over-hand. Science.

[84] Xie P. (2010). Mechanism of processive movement of monomeric and dimeric kinesin molecules. Int. J. Biol. Sci..

[85] Hyeon C., Onuchic J.N. (2007). Mechanical control of the directional stepping dynamics of the kinesin motor. Proc. Natl. Acad. Sci. USA.

[86] Zhang Z., Thirumalai D. (2012). Dissecting the kinematics of the kinesin step. Structure.

[87] Geng Y., Li T., Ji Q., Yan S. (2014). Simulation study of interactions between kinesin’s neck linker and motor domain. Cel. Mol. Bioeng..

[88] Geng Y., Ji Q., Liu S., Yan S. (2014). Initial conformation of kinesin’s neck linker. Chin. Phys. B.

[89] Geng Y., Zhang H., Lyu G., Ji Q. (2017). Initiation mechanism of kinesin’s neck linker docking process. Chin. Phys. Lett..

[90] Hwang W., Lang M.J., Karplus M. (2008). Force generation in kinesin hinges on cover-neck bundle formation. Structure.

[91] Khalil A.S., Appleyard D.C., Labno A.K., Georges A., Karplus M., Belcher A., Hwang W., Lang M.J. (2008). Kinesin’s cover-neck bundle folds forward to generate force. Proc. Natl. Acad. Sci. USA.

[92] Hesse W.R., Steiner M., Wohlever M.L., Kamm R.D., Hwang W., Lang M.J. (2013). Modular aspects of kinesin force generation machinery. Biophys. J..

[93] Rice S.E. (2013). Kinesin-5 seems to step to its own unique tune, but really it’s a cover. Biophys. J..

[94] Qin J., Geng Y., Lü G., Ji Q., Fang H. (2018). Protection-against-water-attack determined difference between strengths of backbone hydrogen bonds in kinesin’s neck zipper region. Chin. Phys. B.

[95] Shi X., Guo S., Wang P., Chen H., Xie P. (2019). All-atom molecular dynamics simulations reveal how kinesin transits from one-head-bound to two-heads-bound state. Proteins.

[96] Milic B., Andreasson J.O.L., Hancock W.O., Block M. (2014). Kinesin processivity is gated by phosphate release. Proc. Natl. Acad. Sci. USA.

[97] Andreasson J.O.L., Milic B., Chen G.Y., Guydosh N.R., Hancock W.O., Block S.M. (2015). Examining kinesin processivity within a general gating framework. eLife.

[98] Mickolajczyk K.J., Deffenbaugh N.C., Arroyo J.O., Andrecka J., Kukura P., Hancock W.O. (2015). Kinetics of nucleotide-dependent structural transitions in the kinesin-1 hydrolysis cycle. Proc. Natl. Acad. Sci. USA.

[99] Humphrey W., Dalke A., Schulten K. (1996). VMD: Visual molecular dynamics. J. Mol. Graph..

[100] Hancock W.O., Howard J. (1999). Kinesin’s processivity results from mechanical and chemical coordination between the ATP hydrolysis cycles of the two motor domains. Proc. Natl. Acad. Sci. USA.

[101] Rosenfeld S.S., Xing J., Jefferson G.M., Cheung H.C., King P.H. (2002). Measuring kinesin’s first step. J. Biol. Chem..

[102] Schief W.R., Clark R.H., Crevenna A.H., Howard J. (2004). Inhibition of kinesin motility by ADP and phosphate supports a hand-over-hand mechanism. Proc. Natl. Acad. Sci. USA.

[103] Crevel I.M., Nyitrai M., Alonso M.C., Weiss S., Geeves M.A., Cross R.A. (2004). What kinesin does at roadblocks: The coordination mechanism for molecular walking. EMBO J..

[104] Sablin E.P., Fletterick R.J. (2004). Coordination between motor domains in processive kinesins. J. Biol. Chem..

[105] Hancock W.O. (2016). The kinesin-1 chemomechanical cycle: Stepping toward a consensus. Biophys. J..

[106] Coy D.L., Hancock W.O., Wagenbach M., Howard J. (1999). Kinesin’s tail domain is an inhibitory regulator of the motor domain. Nat. Cell Biol..

[107] Hackney D.D., Stock M.F. (2000). Kinesin’s IAK tail domain inhibits initial microtubule-stimulated ADP release. Nat. Cell Biol..

[108] Hackney D.D., Stock M.F. (2008). Kinesin tail domains and Mg^2+^ directly inhibit release of ADP from head domains in the absence of microtubules. Biochemistry.

[109] Hackney D.D., Baek N., Snyder A.C. (2009). Half-site inhibition of dimeric kinesin head domains by monomeric tail domains. Biochemistry.

[110] Kaan H.Y., Hackney D.D., Kozielski F. (2011). The structure of the kinesin-1 motor-tail complex reveals the mechanism of autoinhibition. Science.

[111] Kuznetsov S.A., Gelfand V.I. (1986). Bovine brain kinesin is a microtubule-activated ATPase. Proc. Natl. Acad. Sci. USA.

[112] Hackney D.D. (1988). Kinesin ATPase: Rate-limiting ADP release. Proc. Natl. Acad. Sci. USA.

[113] Kikkawa M. (2008). The role of microtubules in processive kinesin movement. Trends Cell Biol..

[114] Mori T., Vale R.D., Tomishige M. (2007). How kinesin waits between steps. Nature.

[115] Tomishige M., Vale R.D. (2000). Controlling kinesin by reversible disulfide cross-linking: Identifying the motility-producing conformational change. J. Cell Biol..

[116] Hackney D.D., Stock M.F., Moore J., Patterson R.A. (2003). Modulation of kinesin half-site ADP release and kinetic processivity by a spacer between the head groups. Biochemistry.

[117] Rosenfeld S.S., Fordyce P.M., Jefferson G.M., King P.H., Block S.M. (2003). Stepping and stretching. How kinesin uses internal strain to walk processively. J. Biol. Chem..

[118] Uemura S., Ishiwata S. (2003). Loading direction regulates the affinity of ADP for kinesin. Nat. Struct. Biol..

[119] Hyeon C., Onuchic J.N. (2007). Internal strain regulates the nucleotide binding site of the kinesin leading head. Proc. Natl. Acad. Sci. USA.

[120] Yildiz A., Tonishige M., Gennerich A., Vale R.D. (2008). Intramolecular strain coordinates kinesin stepping behavior along microtubules. Cell.

[121] Clancy B.E., Behnke-Parks W.M., Andreasson J.O., Rosenfeld S.S., Block S.M. (2011). A universal pathway for kinesin stepping. Nat. Struct. Mol. Biol..

[122] Hariharan V., Hancock W.O. (2009). Insights into the mechanical properties of the kinesin neck linker domain from sequence analysis and molecular dynamics simulations. Cell. Mol. Bioeng..

[123] Shastry S., Hancock W.O. (2011). Interhead tension determines processivity across diverse N-terminal kinesins. Proc. Natl. Acad. Sci. USA.

[124] Guydosh N.R., Block S.M. (2006). Backsteps induced by nucleotide analogs suggest the front head of kinesin is gated by strain. Proc. Natl. Acad. Sci. USA.

[125] Dogan M.Y., Can S., Cleary D.B., Purde V., Yildiz A. (2015). Kinesin’s front head is gated by the backward orientation of its neck linker. Cell Rep..

[126] Sosa H., Peterman E.J.G., Moerner W.E., Goldstein L.S.B. (2001). ADP-induced rocking of the kinesin motor domain revealed by single-molecule fluorescence polarization microscopy. Nat. Struct. Biol..

[127] Toprak E., Yildiz A., Hoffman M.T., Rosenfeld S.S., Selvin P.R. (2009). Why kinesin is so processive. Proc. Natl. Acad. Sci. USA.

[128] Cross R.A., McAinsh A. (2014). Prime movers: The mechanochemistry of mitotic kinesins. Nat. Rev. Mol. Cell Biol..

[129] Friel C.T., Howard J. (2011). The kinesin-13 MCAK has an unconventional ATPase cycle adapted for microtubule depolymerization. EMBO J..

[130] Henrichs V., Grycoval L., Barinka C., Nahacka Z., Neuzil J., Diez S., Rohlena J., Braun M., Lansky Z. (2020). Mitochondria-adaptor TRAK1 promotes kinesin-1 driven transport in crowded environments. Nat. Commun..

[131] Konishi Y., Setou M. (2009). Tubulin tyrosination navigates the kinesin-1 motor domain to axons. Nat. Neurosci..

[132] Dunn S., Morrison E.E., Liverpool T.B., Molina-Paris C., Cross R.A., Alonso M.C., Peckham M. (2008). Differential trafficking of Kif5c on tyrosinated and detyrosinated microtubules in live cells. J. Cell Sci..

[133] Sirajuddin M., Rice L.M., Vale R.D. (2014). Regulation of microtubule motors by tubulin isotypes and post-translational modifications. Nat. Cell Biol..

